# Quorum-Sensing Signal Disperses Bacteria from Biofilms

**DOI:** 10.1289/ehp.120-a420

**Published:** 2012-11-01

**Authors:** Carol Potera

**Affiliations:** Carol Potera, based in Montana, has written for *EHP* since 1996. She also writes for *Microbe*, *Genetic Engineering News*, and the *American Journal of Nursing*.

Biofilms are complex bacterial communities that range from bioluminescent pools in oceans to plaque buildup on teeth. These communities use quorum-sensing signals to modify their behaviors for optimal resilience against potential threats. Now researchers who study biofilms that cohabitate with marine sponges have discovered a quorum-sensing signal that controls the formation of the flagellum, a corkscrew-like appendage that rotates and allows bacteria to swim away from a biofilm.^1^

“It was a total surprise,” says Russell Hill, director of the University of Maryland’s Institute of Marine and Environmental Technology in Baltimore. Generally, bacterial signaling acts in the opposite way: Bacteria use flagella to swim into areas where they accumulate to high concentrations. Then quorum sensing causes them to lose motility and become fixed in place. Typically, Hill explains, “They *form* a biofilm rather than *leave* a biofilm.”

Biofilms strongly adhere to surfaces and resist antibiotics and other antimicrobial agents. They can cause many problems, including contamination of catheters, heart valves, contact lenses, and other medical devices.^2^ They also infect the lungs of patients with cystic fibrosis^3^ and cause periodontal disease^4^ and otitis media.^5^ By one estimate, up to 60% of all human bacterial infections in developed countries are caused by biofilms.^2^ In industrial settings, biofilms corrode pipes, clog filters, foul ship hulls, and contaminate food-processing equipment.^2^

Hill and colleagues at Indiana University Bloomington and the University of Colorado Denver focused on *N*-acylhomoserine lactone quorum-sensing signaling molecules in the bacterium *Ruegeria* KLH11. This microbe lives symbiotically within the sponge *Mycale laxissima*, which the investigators collected from the sea near Key Largo, Florida. Marine sponges harbor complex and diverse bacterial communities, which can make up as much as 40% of the sponge’s biomass.^6^ “Sponges provide an excellent model system for understanding complex symbiosis and quorum sensing,” says Hill.

The researchers genetically analyzed *Ruegeria* KLH11 and identified two sets of regulatory genes involved in quorum sensing, *SsaRI* and *SsbRI*. Further experiments with knockout mutants showed that *SsaI* produces quorum-sensing signals that control the formation of flagella and motility. The knockout mutants that lack flagella not only could not swim but also built biofilms up to twice the size of those built by normal *Ruegeria* KLH11. The authors suggest that the mutants’ loss of motility and inability to leave biofilms likely contributed to the increased biofilm formation.^1^

**Figure:**
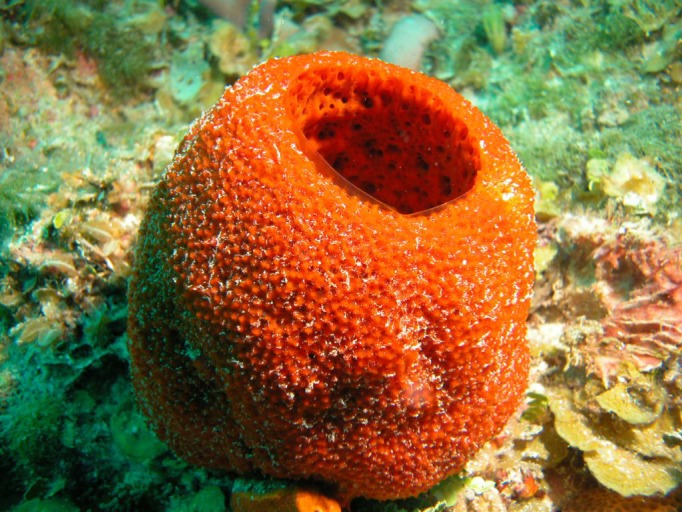
The marine sponge *Mycale laxissima* hosts the bacterium Ruegeria KLH11. Sven Zea/http://www.spongeguide.org/speciesinfo.php?species=14

The findings indicate that *Ruegeria* KLH11 uses quorum sensing in a previously unsuspected way, to activate “a dispersal mechanism that promotes the uniform distribution of these bacteria in the local environment when the population becomes too dense,” says coauthor Clay Fuqua, a professor of biology at Indiana University Bloomington. This process may have evolved to help maintain healthy symbiotic bacterial communities.

The latest findings about bacterial communication “may be important in understanding how bacteria become pathogenic in humans or form biofilms on teeth or medical devices,” says Hill. However, he cautions that it’s much too early to extrapolate the results to solving any of these biofilm problems. “We are building a basic knowledge that may eventually have practical applications, but there is no clear path to a practical application just yet,” he says.

The detailed mechanics of the quorum-sensing system described by Hill and colleagues “are of immense interest to researchers in far-reaching fields, including biotechnology, medicine, microbial ecology, microbial communication, and biofilm formation,” says Alison Buchan, an associate professor of microbiology at the University of Tennessee, Knoxville. They are a reminder that bacterial quorum-sensing systems and the microbial physiologies they influence are varied and complex, particularly for the control of biofilm formation. “Quorum sensing can either promote or inhibit biofilm formation, depending on the bacterial species,” Buchan says. “This suggests that there is no one silver bullet or universal approach to inhibit all bacterial biofilms.”

Future studies of the microbial ecology of biofilms that live with sponges could benefit coral reefs as well as humans. Sponges are an important component of coral reefs, and diseases of sponges are increasing, perhaps due to rising water temperatures.^7^ “It could be that biofilms are more sensitive to temperature fluctuations than their host sponges, whose well-being depends on maintaining healthy microbial neighbors. So it’s important to understand in detail the bacterial communities associated with sponges,” says Hill.
